# Catastrophic Tibial Baseplate Fracture in the TC-PLUS™ Total Knee System: A Case Report and Literature Review

**DOI:** 10.7759/cureus.94665

**Published:** 2025-10-15

**Authors:** Fotios Panagopoulos, Vasileios Athanasiou, Vasileios Giannatos, Michail Kroustalakis, Panagiotis Antzoulas, John Gliatis

**Affiliations:** 1 Department of Orthopaedics and Traumatology, General University Hospital of Patras, Patras, GRC

**Keywords:** cantilever effect, catastrophic tibial baseplate fracture, primary total knee arthroplasty, total knee arthroplasty implants, total knee revision arthroplasty

## Abstract

The number of patients having total knee arthroplasty (TKA) is advancing rapidly. The outcome of this rapid development is an increase in revision TKA (rTKA). A rare indication for rTKA is implant failure, most notably fracture of the tibial baseplate. The pathway leading to the fracture is based upon the theory of the cantilever effect between the supported and unsupported tray. This phenomenon can be a result of chronic osteolysis due to malalignment of implants and insufficient ligament balance, or in shorter postoperative time due to undersizing of the implants in combination with false placement, which leads to inadequate cortical support. This paper presents a case of tibial tray fracture occurring 17 years after primary TKA, managed with a hinged revision, along with a literature review of this rare complication.

## Introduction

Total knee arthroplasty (TKA) is the most frequently performed joint replacement procedure globally, due to its well-documented clinical efficacy and cost-effectiveness in treating end-stage osteoarthritis [1]. To prevent subsidence and loosening, as well as to improve the distribution of weight-bearing forces, a major advancement in knee replacement systems was the introduction of the tibial baseplate [2]. Fractures of the tibial component in TKA are rare [3]. Early reports documented such fractures in older-generation TKA designs with metal tibial baseplates. However, with continuous advancements in implant materials and design, these events have become increasingly uncommon [3]. Fracture of the metal tibial component is a severe complication that necessitates complex revision arthroplasty, particularly in cases of aseptic loosening of the medial compartment, where focal proximal tibial bone loss creates differential support of the baseplate, resulting in cantilever bending at the junction between supported and unsupported bone, which in turn generates abnormal stress concentrations that predispose to fatigue failure of the implant and ultimately lead to fracture, progressive varus collapse, accelerated polyethylene wear, and extensive bone loss that further complicate revision surgery [3,4].

We present a case of tibial tray fracture occurring 17 years after primary TKA, managed with a hinged revision system due to underlying osteolysis of the medial compartment. This report aims to highlight this rare complication and to review the current literature on the subject.

## Case presentation

An 84-year-old female patient presented to our outpatient clinic with symptoms of joint effusion, pain in extension, and concomitant instability. Her medical history was significant for hypertension and metabolic syndrome, including type 2 diabetes mellitus and dyslipidemia, both under medical management. Surgically, she had undergone bilateral TKA 17 years prior at a private hospital. The type of the loose prosthesis is TC-PLUS™ Total Knee System (Smith & Nephew, London, UK).

**Figure 1:**
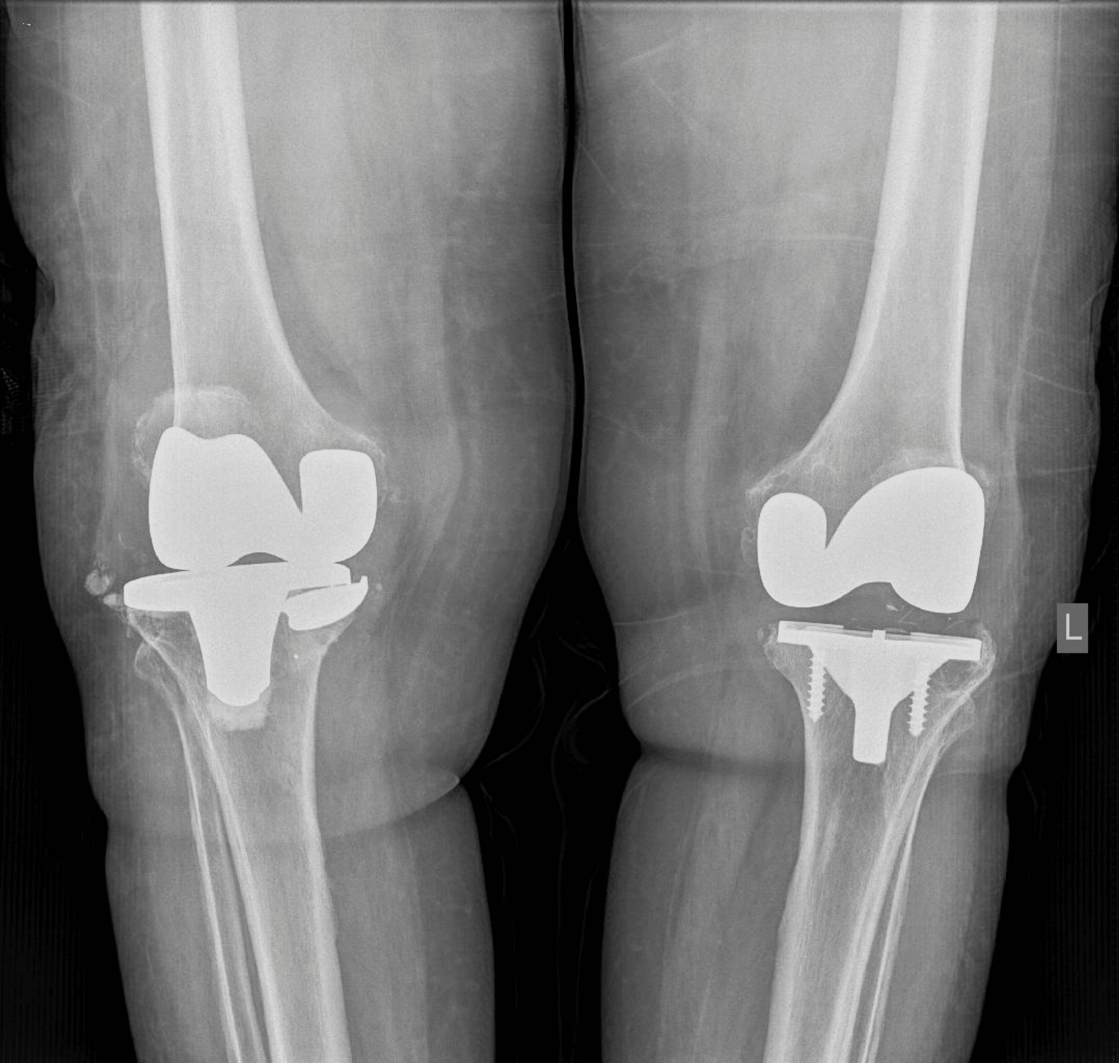
Preoperative radiographs obtained at the outpatient clinic, demonstrating a fracture of the tibial tray in the right knee. Note the radiolucent area in the tibial epiphysis, indicative of osteolysis.

The patient reported symptom onset nine months prior to consultation, with no history of preceding trauma or falls during that period. Clinical examination revealed joint effusion, erythema, and a painful arc of motion accompanied by crepitus of the affected limb, as well as medial laxity under stress testing. Standard radiographic evaluation confirmed the presence of a tibial tray fracture (Figure 1).

One of the key radiographic findings was significant osteolysis of the medial plateau in both knees. Laboratory tests were negative for inflammatory markers (Table 1). Synovial fluid analysis revealed (Table 2) [5].

**Table 1:** Preoperative blood test, negative variables for septic arthritis. WBC: White blood cells, PMN: Polymorphonuclear blood cells, ESR: Erythrocyte sedimentation rate, CRP: C-reactive proteins

Blood examination results	Patient pre-op	Normal values
WBC (K/μλ)	5	4.0-11
PMN (%)	54	50-70
ESR (mm)	17	0-20
Crp (mg/dL)	0.6	<0.8

**Table 2:** Synovial fluid examination.

Synovial fluid analysis	Patient pre-op	Normal values
Serum appearance	Red colour	Clear
Ph	7.2	7.30-7.55
Total cell count	20.000	-
WBC	1000	<2.000 cells/μL
Erythrocytes	19.000	0 cells/μL
Gram-stain	Negative	-
Z-N stain	Negative	-

**Figure 2:**
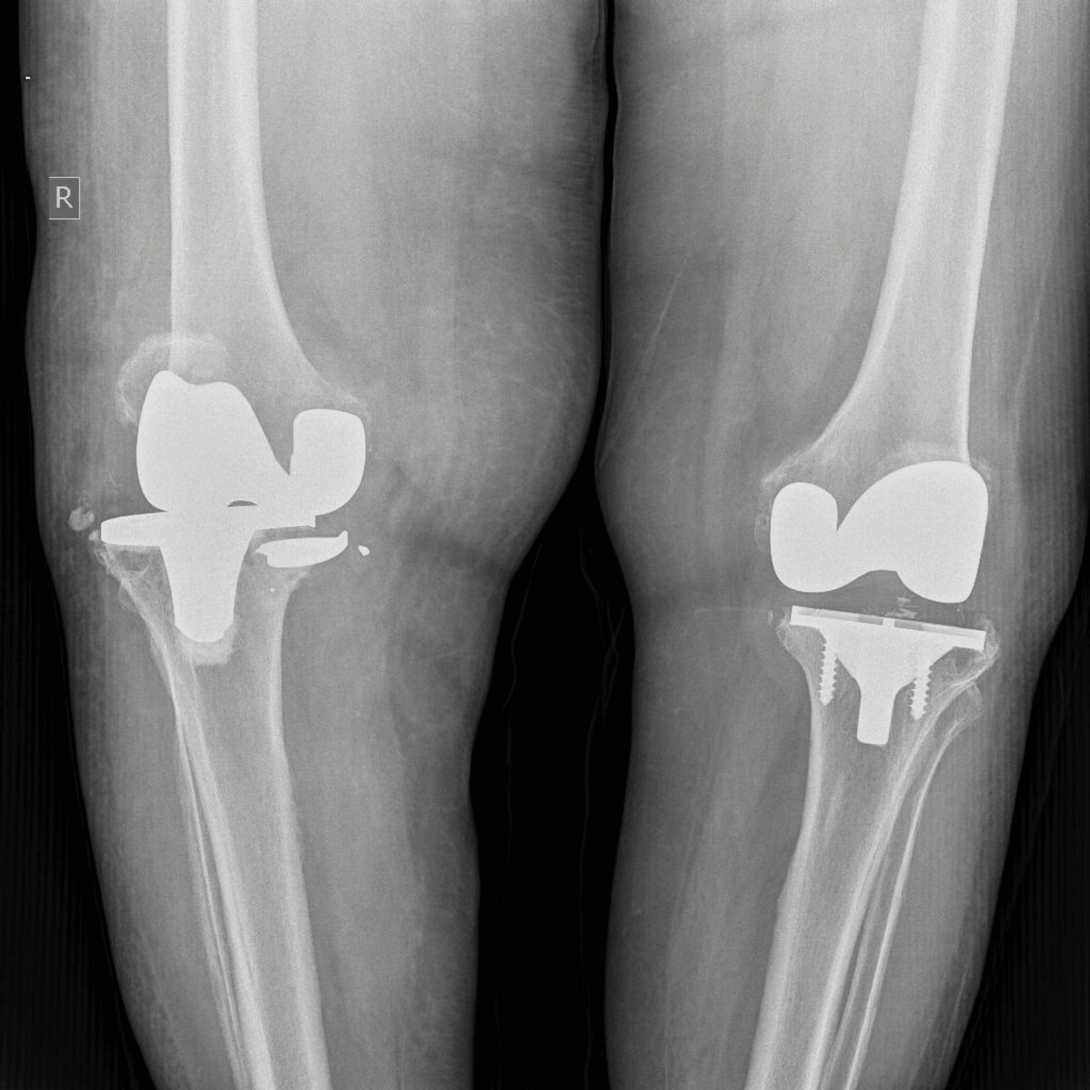
Radiographs were obtained at the outpatient clinic three months after the initial assessment, during preoperative planning. Note the subsidence of the fractured segment of the tibial tray.

Blood and synovial fluid cultures were negative for infection. After excluding septic arthritis, the patient was scheduled for revision TKA. Preoperative radiographs demonstrated progression of osteolysis in the lateral compartment, accompanied by subsidence of the tibial tray (Figure 2).

**Figure 3:**
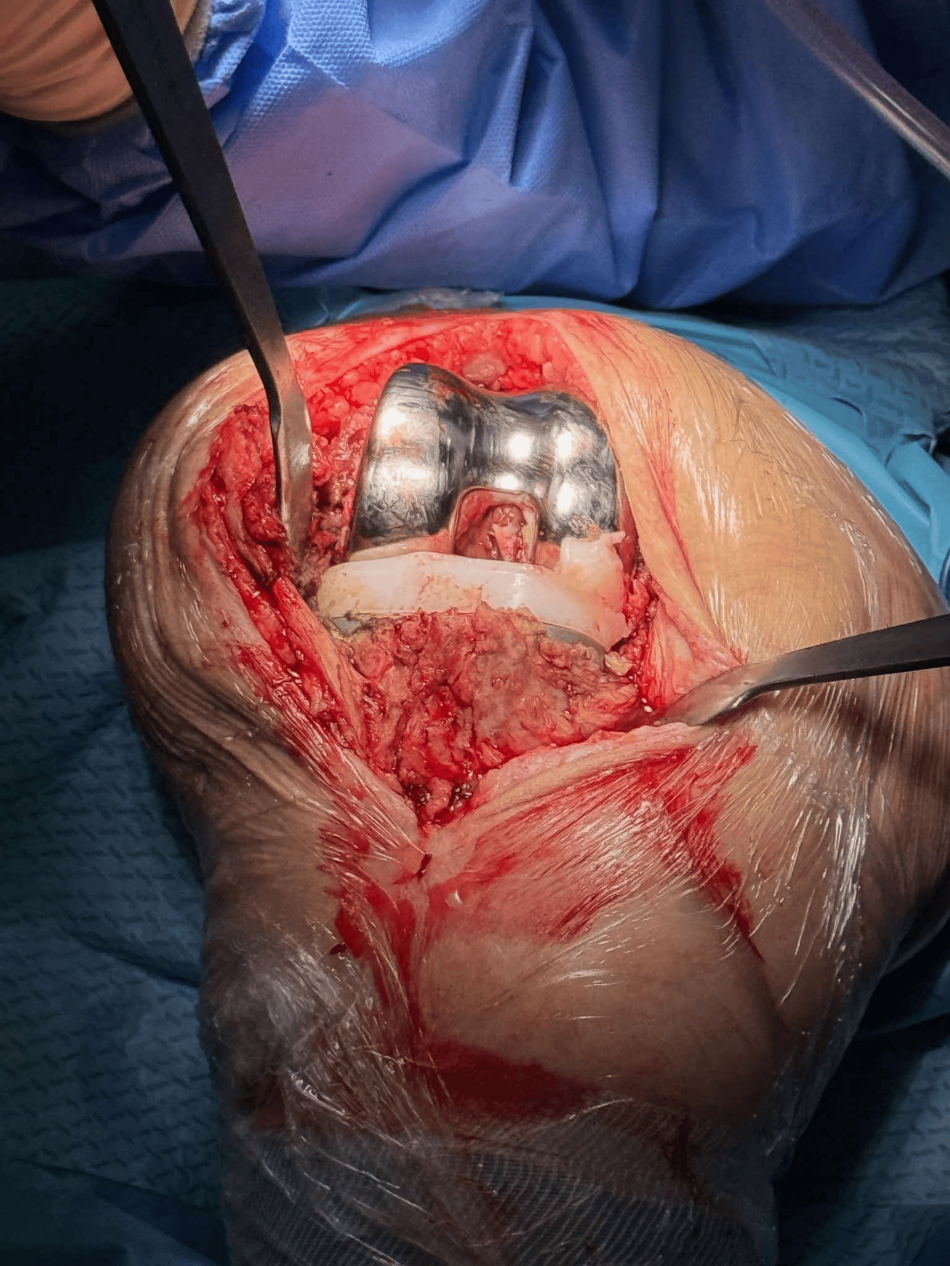
Intraoperative image demonstrating the polyethylene insert tear.

**Figure 4:**
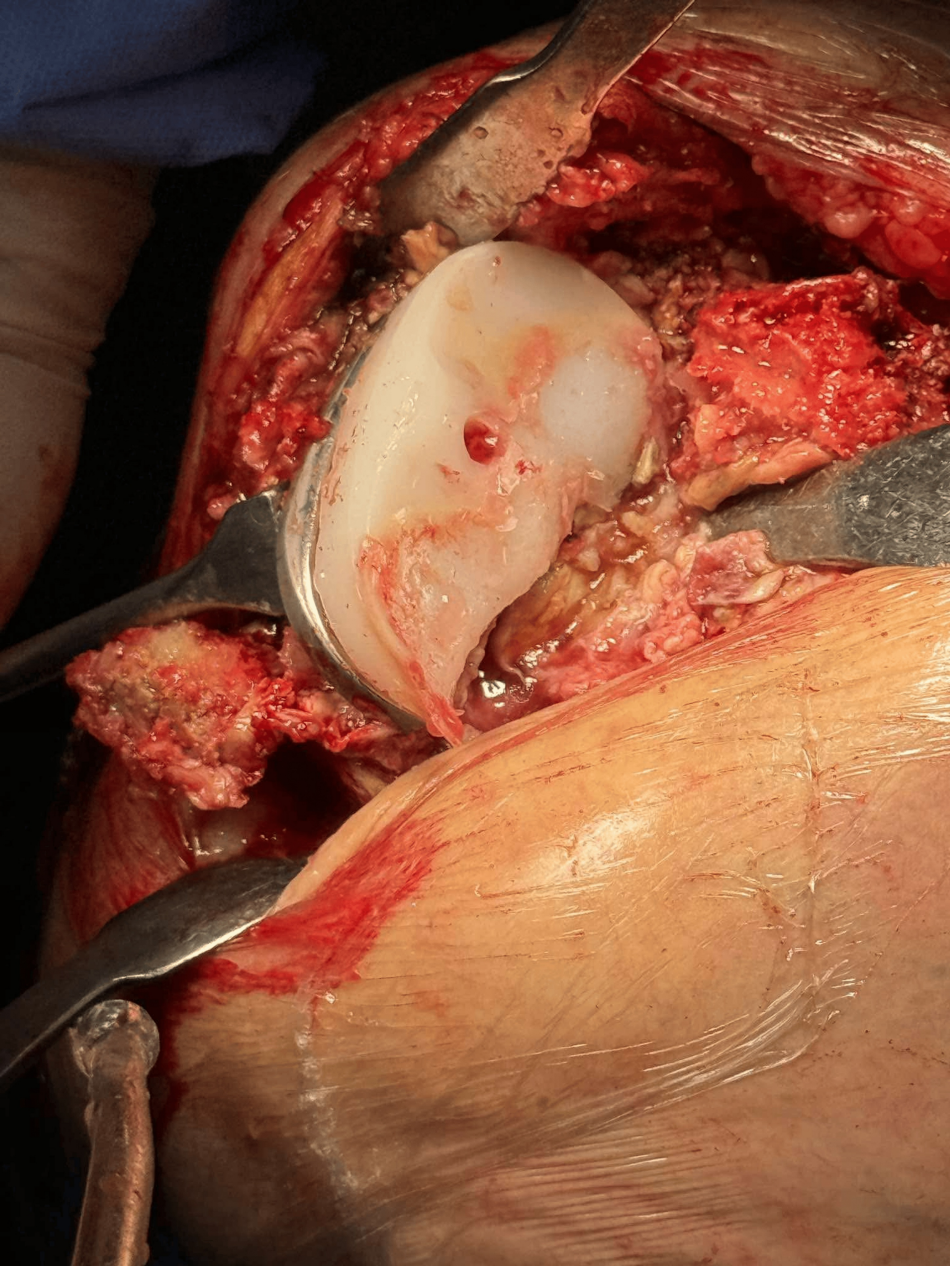
Intraoperative image following removal of the femoral component.

**Figure 5:**
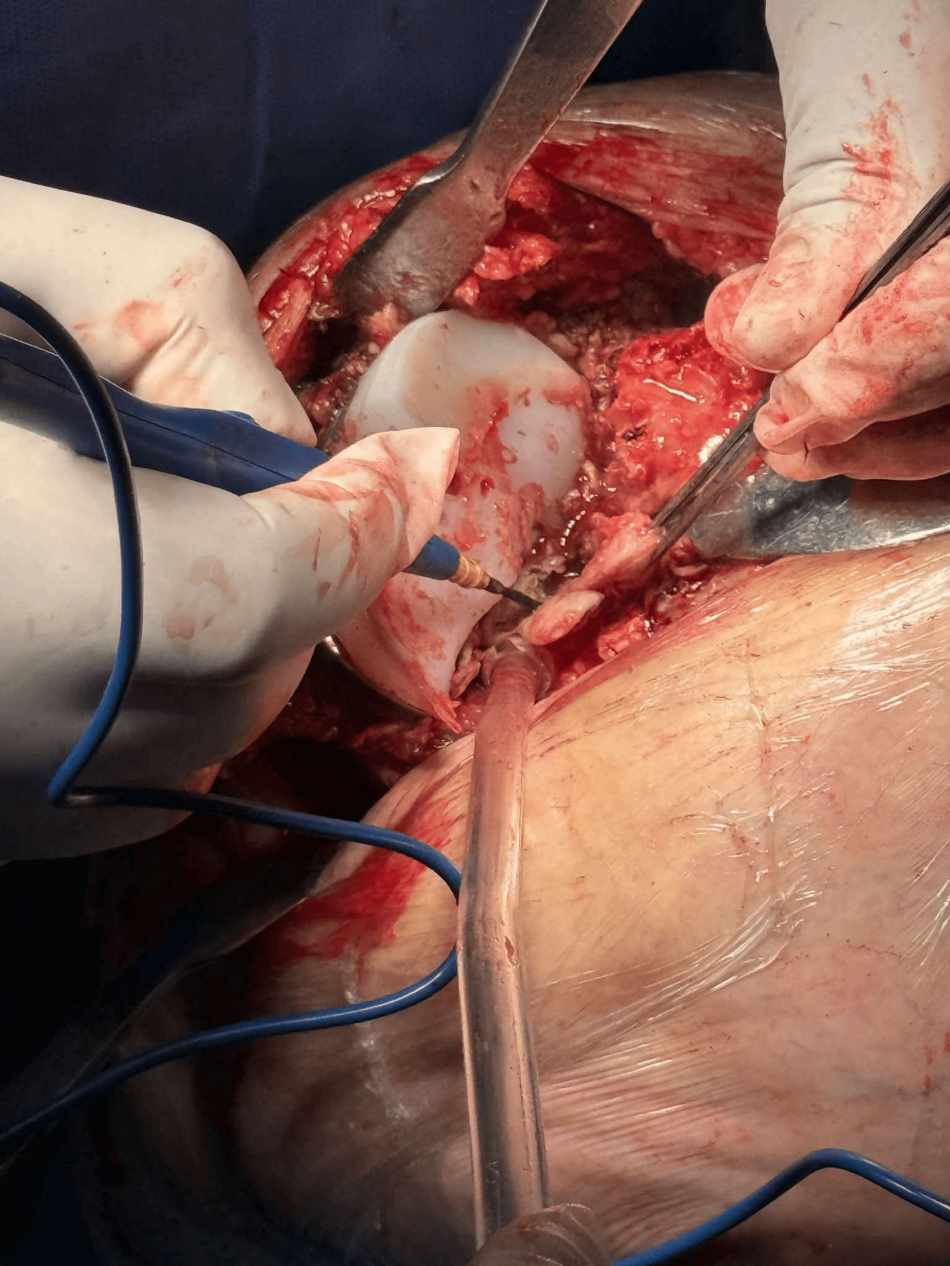
Excision of soft tissue attachments and release of the posterior cruciate ligament (PCL).

The previous skin incision was utilized, with a standard dissection through the skin and subcutaneous tissue, followed by a medial parapatellar approach for exposure of the knee joint. Intraoperatively, a tibial tray fracture was identified along with a concomitant polyethylene insert fracture on the posteromedial aspect (Figures 3-5).

**Figure 6:**
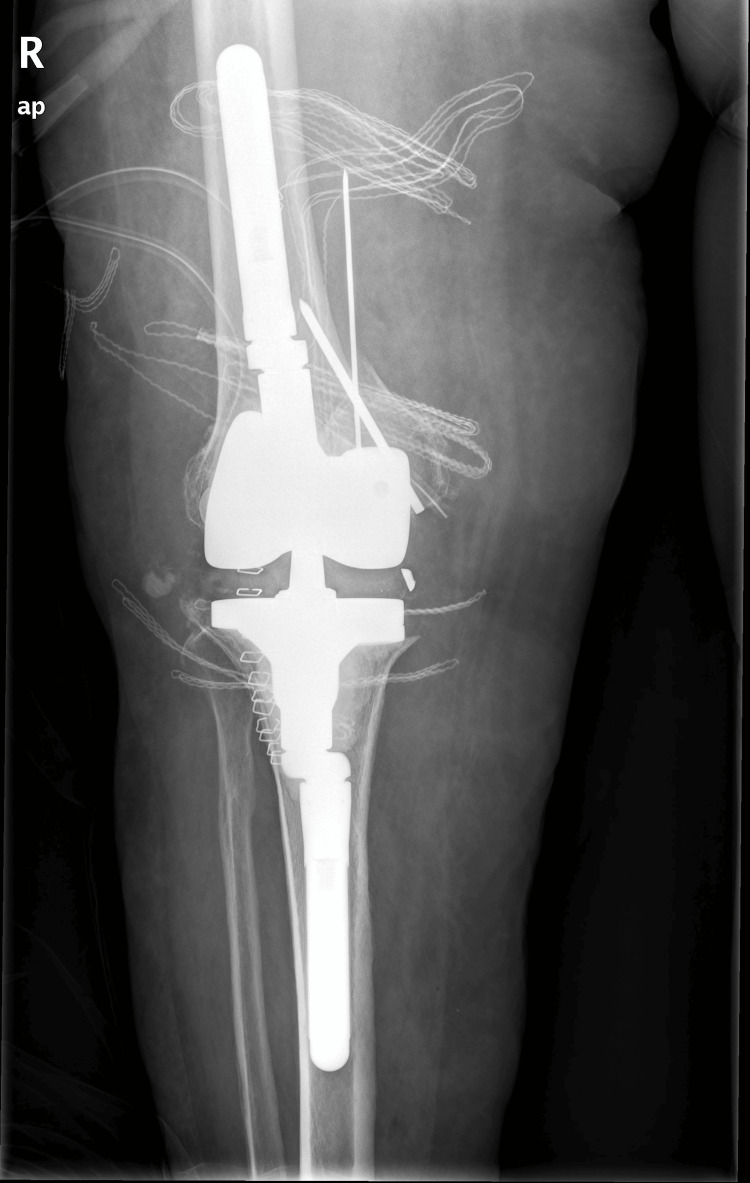
Immediate postoperative AP radiograph of a hinged total knee arthroplasty showing femoral and tibial offsets and pins securing the medial femoral condyle.

**Figure 7:**
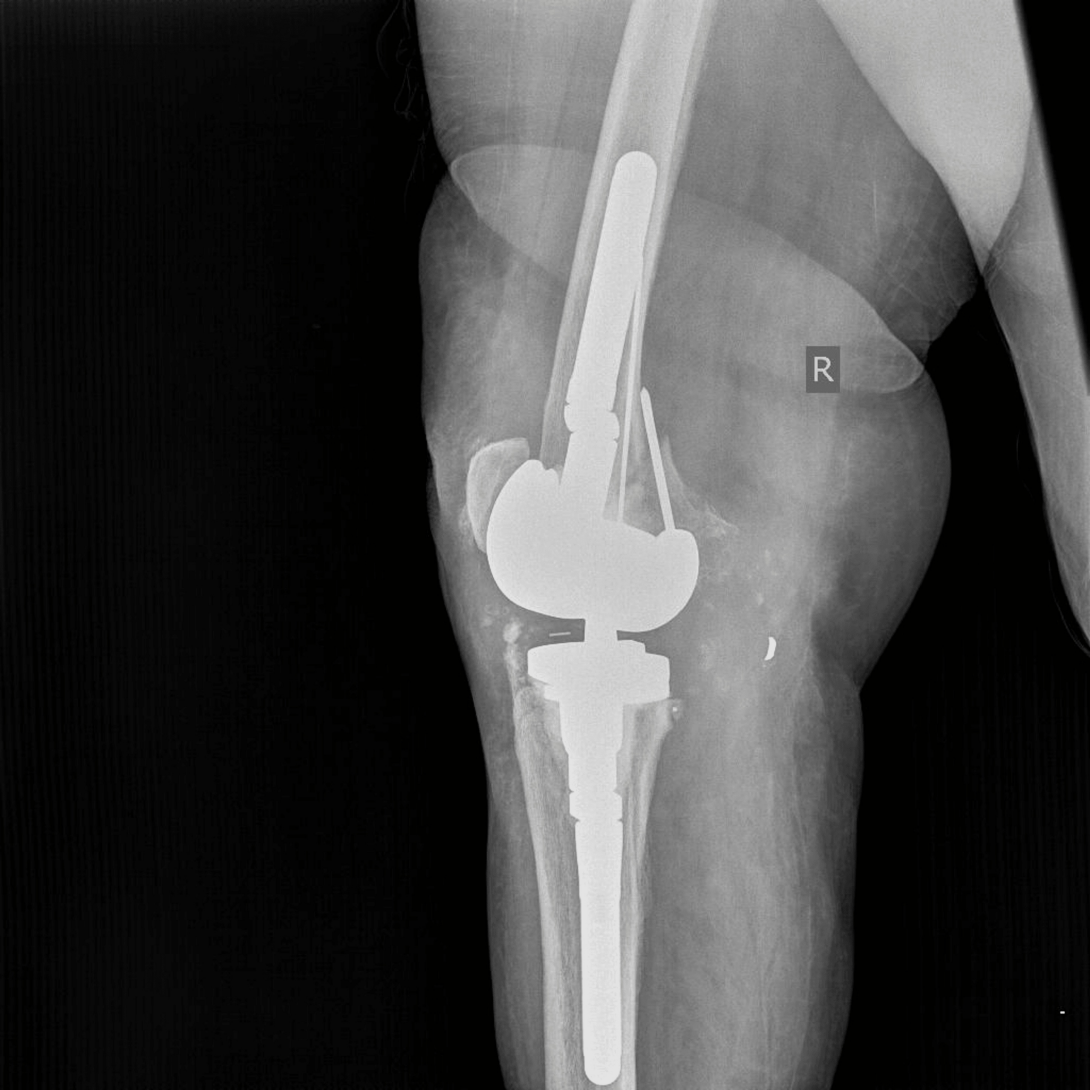
Immediate postoperative (L) radiograph of a hinged total knee arthroplasty showing femoral and tibial offsets and pins securing the medial femoral condyle.

Following implant removal using a surgical saw, bone loss was observed on the medial tibial plateau; however, it was confined to the epiphysis, corresponding to type 2A according to the Anderson Orthopaedic Research Institute (AORI) classification [5]. During removal of the femoral component, an intraoperative fracture of the medial femoral condyle occurred, which was subsequently stabilized with a pin (Figures 6-7).

Varus/valgus stress testing revealed medial collateral ligament (MCL) insufficiency with the absence of an endpoint. The MCL insufficiency was reaffirmed by the finding of sutures in the mass of the ligament fibers during the rTKA.Considering the bone and soft tissue deficiencies, a MUTARS® GenuX® MK Revision Knee System (Implantcast, Inc., Buxtehude, Germany) was implanted, comprising a size no. 2 tibial baseplate with a 15 mm metaphyseal stem, augmented medially with a 5 mm wedge. The femoral component was size no. 2, also augmented medially by 5 mm, coupled with a 17.5 mm polyethylene insert. The wound was closed in layers, and tissue samples from areas suggestive of necrosis were sent for culture. The postoperative hospital stay was uneventful and brief. Cultures of tissue and bone obtained intraoperatively were negative for infection. On the last follow-up, one year post-op op, the patient had an excellent range of motion. The specific data after one year showed 79 points in the Knee Society Score, as the patient reported major relief from pain with mild symptoms of discomfort when walking up the stairs, an extension lag of 5 degrees, no flexion contractures, and an excellent range of motion of 0-95 degrees. No major instability was noticed during follow-up. The reported functional report was 80 because of the discomfort during walking the stairs.

## Discussion

The primary causes of rTKA include infection, aseptic loosening, and instability, with varying reported frequencies [6]. A rare cause is implant fracture, specifically fracture of the tibial baseplate, with an incidence reported between 0.13% and 0.3% [7].

The first report of this phenomenon was published by Scott et al. in 1984, followed by several additional studies over the subsequent decade. These fractures were primarily attributed to manufacturing defects, rendering the implants susceptible to fatigue failure [8,9]. To date, the retrieval analysis by Chatterji et al. remains the only systematic investigation of implant fractures in TKA, demonstrating a strong correlation between the site of tibial baseplate failure and the presence of underlying proximal tibial bone loss [10].

Callaghan et al. proposed a three-stage model for fatigue fractures, describing initiation, propagation, and sudden fracture. They attributed these fractures to osteolysis of the metaphyseal cancellous bone caused by polyethylene wear and unbalanced medial loading, which creates a cantilever effect between the supported and unsupported regions of the tibial baseplate [11,12]. Mineta et al. reported that, after 16 years, varus malalignment of the tibial tray combined with improper ligament balance led to polyethylene wear, aseptic loosening, and subsequent fracture [2]. Additionally, Callaghan et al. described a case involving a patient with elevated body mass index and chronic oral corticosteroid use, who developed aseptic loosening secondary to necrotic bone [12].

As previously mentioned, the patient had an elevated body mass index (BMI), which is an independent risk factor for aseptic loosening. Cankaya et al. reported a case in which increased BMI combined with pre-existing varus malalignment led to implant fracture, attributed in part to inadequate postoperative follow-up after the index procedure [13].

To the best of our knowledge, this is the first reported case of a fracture involving the TC-PLUS™ Total Knee System. This implant represents a contemporary prosthesis design, free from historical design limitations such as an extensive posterior cut-out for the cruciate ligament, thin baseplate thickness, suboptimal material selection (with cobalt-chrome alloys demonstrating higher failure rates compared to titanium), and unfavorable geometric configuration [11].

The significance of component positioning in implant failure has been well established. In this case, preoperative radiographs of the native arthritic knee were not available. The left limb demonstrated significant varus deformity secondary to osteolysis, accompanied by concurrent osteolysis of the medial femoral condyle.

The proposed mechanism suggests that varus malalignment resulted in excessive stress on the medial femoral and tibial compartments. Consequently, the medial femoral condyle fractured during removal of the primary TKA, while the tibial tray fractured during ambulation. Due to the tenderness of the affected limb, an accurate anteroposterior (AP) radiograph could not be obtained prior to surgery; however, varus alignment was confirmed intraoperatively by extensive erosion of the medial polyethylene insert surface [14].

Sepehri et al. proposed an additional mechanism for tibial baseplate fracture, attributing it to inadequate tibial coverage in a specific region combined with an undersized tibial tray and lateral positioning relative to the tibial tubercle. This configuration resulted in insufficient cortical support and a subsequent baseplate fracture three years postoperatively [15]. Lam et al. further expanded on these concepts in the context of cementless fixation techniques [16]. Table 3 presents the literature review for this study.

**Table 3:** Literature review. rTKA: Revision total knee arthroplasty

Study	Time of fracture after index surgery	Type of prosthesis	Cause	Treatment
Scott et al., 1984 [7]	Not specified	Early design TKA, with a metal-backed tibial tray	Fatigue fracture of the metallic tibial tray due to long-term varus malalignment	rTKA
Cook et al., 1991 [9]	28 months and 35 months	Porous coated titanium alloy PFC Johnson and Johnson	Fatigue failure due to cyclic loading and thin thickness	rTKA
Altıntaş et al., 1999 [8]	6 years	Miller–Galante II total knee arthroplasty (TKA)	Osteolysis, aseptic loosening, fatigue fracture because of neutral anatomic rotation of the femoral component, baseplate smaller than the plateau	rTKA with bone graft
Mineta et al., 2017 [2]	16 years	Miller–Galante II total knee arthroplasty (TKA)	Polyethylene wear, osteolysis led to the cantilever effect and fracture of the plate	rTKA with a long-stem tibial prosthesis and bone grafting
Chatterji et al., 2005 [10]	Mean 10.8 months	10 Kinematic, 6 Miller-Galante (M-G), 5 Porous Coated Anatomic (PCA), and 4 Ortholoc II	Polyethylene wear, osteolysis, aseptic loosening, and predisposing design flaws	Not specified
Cankaya et al., 2014 [13]	11 years after the primary TKA	Not specified	Asymmetric loading, osteolysis with a neglected tibial baseplate at the consultation	rTKA with bone grafts and augments for bone loss
da Palma et al., 2015 [14]	9 and 10 years each after index procedure	PCA (Howmedica®)	Varus malalignment of the components due to insufficient ligament balancing. One patient was obese	rTKA with a long-stem tibial prosthesis, augmented and bone grafting
Callaghan et al., 2018 [12]	1 year	PFC Sigma design (DePuy, Warsaw, IN)	Necrotic bone due to asymmetric loading and corticosteroid use	rTKA with bone grafts and augments for bone loss
Scully et al., 2019 [11]	4 years	Zimmer NexGen tibial baseplate	component malpositioning, inadequate component sizing, and elevated patient body weight	rTKA with bone grafts and augments for bone loss
Sepehri et al., 2020 [15]	3 years	NexGen Posterior Stabilized LPS-Flex system (Zimmer-Biomet, Warsaw, IN)	Lack of cortical structural support, false positioning of the components, and prosthesis overload from high BMI and activity level	rTKA with bone grafts augments and a cone for bone loss
Lam et al., 2022 [16]	3 years	Triathlon Tritanium Tibial Baseplate; Stryker Orthopedics, Mahwah, NJ	Design weakness, high activity level	RTKA

## Conclusions

This case report describes a female patient who underwent rTKA due to a tibial tray fracture occurring 17 years after the primary implantation. Malpositioning of the implants combined with elevated BMI was identified as a contributing factor leading to osteolysis and subsequent implant failure. To our knowledge, this represents the first reported case of failure involving the TC-PLUS™ Total Knee System. Further prospective studies are warranted to identify additional risk factors associated with this rare complication.

In conclusion, the current literature suggests that tibial base plate fractures result from two distinct pathways. The first and most common rationale supports the theory that either insufficient ligament balancing, varus position of the tibial component, excessive loading due to high BMI index, sizing mismatch of the components, or high activity level leads to rapid polyethylene wear, which causes osteolysis, resulting in increased bending forces between the supported and the unsupported parts, leading to fracture. The second rationale causes fractures early after the index procedure. This theory supports that inadequate tibial coverage, due to implant undersizing or malrotation of the implant, produces insufficient cortical support, and predisposes to implant fractures.
